# Human respiratory syncytial virus regulates the expression of interferon-stimulated genes through modulation of fibrillarin

**DOI:** 10.3389/fcimb.2026.1706028

**Published:** 2026-04-15

**Authors:** José Manuel Ulloa-Aguilar, Victor Javier Cruz-Holguin, Yazmín Rocío Benítez-Zeferino, Edgar Rodrigo Guzmán-Bautista, Julio García-Cordero, Luis Adrián De Jesús-González, Julio Angel Vázquez-Martínez, Edgar Ricardo Vázquez-Martínez, Luis Herrera-Moro Huitron, Alfredo Mosqueda-Gracida, Monica Viveros-Rogel, Moises Vergara-Mendoza, Roxana Uri Miranda-Labra, Moises León-Juárez

**Affiliations:** 1 Laboratorio de Virología Perinatal y Diseño Molecular de Antigenos y Biomarcadores, Departamento de Inmunobioquimica, Instituto Nacional de Perinatología, Mexico City, Mexico; 2 Posgrado en Biología Experimental, Divsión de Ciencias biologicas y de la Salud (DCBS), Universidad Autónoma Metropolitana- Iztapalapa, Mexico City, Mexico; 3 Instituto de Investigación Sobre la Salud Pública, Universidad de la Sierra Sur, Miahuatlan de Porfirio Díaz, Oaxaca, Mexico; 4 Departamento de Biomedicina Molecular, Centro de Investigación y de Estudios Avanzados del Instituto Politécnico Nacional (CINVESTAV-IPN), Mexico City, Mexico; 5 Unidad de Investigación Biomédica de Zacatecas, Instituto Mexicano del Seguro Social, Zacatecas, Mexico; 6 Department of Immunology, H. Lee Moffitt Cancer Center, Tampa, FL, United States; 7 Unidad de Investigación en Reproducción Humana, Instituto Nacional de Perinatología - Facultad de Química, Universidad Nacional Autónoma de México, Mexico City, Mexico; 8 Departamento de Infectología, Instituto Nacional de Ciencias Médicas y Nutrición Salvador Zubirán, Mexico City, Mexico; 9 Departamento de Ciencias de la Salud, Universidad Autónoma Metropolitana, Unidad Iztapalapa, Mexico City, Mexico

**Keywords:** ARIS, fibrillarin, innate immunity, ISGs, nucleolus, RSV

## Abstract

**Introduction:**

Human respiratory syncytial virus (RSV) is one of the main viral agents associated with the development of acute respiratory infections (ARIs), particularly during infancy and early childhood. RSV vaccine have recently been approved, however, are currently limited to older adults and pregnant women, with no approval for young children. In the absence of broadly effective and accessible preventive or therapeutic options for this vulnerable population, understanding the biology of RSV represents a critical alternative strategy. While several viral proteins have been reported to regulate the expression of interferon-stimulated genes (ISGs) to evade the host antiviral immune response, recent studies have shown that some viruses can also recruit host cellular proteins to facilitate their replication or modulate antiviral pathways. In this context, the nucleolus, and its resident proteins, such as fibrillarin (FBL), have been suggested to play a role in the regulation of inflammatory responses and in the activation of genes involved in early antiviral defense mechanisms.

**Methods:**

To analyze FBL expression under viral infection conditions, immunofluorescence assays (IFA) and Western blot (WB) analyses were performed. The effects of FBL depletion were evaluated using WB, IFA, RT-qPCR, and lytic plaque assays. Three experimental conditions were established: uninfected A549 cells (mock), RSV-infected cells, and RSV-infected cells with FBL knockdown. To determine the relationship between FBL and interferon-stimulated gene (ISG) expression, RT-qPCR assays were performed to quantify the expression levels of selected ISGs, including OAS1, OAS2, IFIT3, PKR, and RIG-I. Additionally, FBL-knockdown cells were transfected with a GFP-FBL construct to restore FBL expression, and the recovery of RSV infection was evaluated by IFA, RT-qPCR, and plaque-forming unit (PFU) assays. Moreover, the downregulation of ISG expression in cells with restored GFP-FBL was assessed by RT-qPCR. Finally, p53 knockdown assays were performed to evaluate changes in FBL expression and the reduction of RSV infection, as determined by WB.

**Results:**

RSV infection was found to induce FBL expression at early stages of infection in A549 cells. Additionally, our data suggest that FBL suppresses the expression of interferon-stimulated genes (ISGs). Conversely, silencing of FBL significantly reduced RSV infection. Importantly, this reduction in viral replication was associated with increased ISG expression in FBL-deficient A549 cells upon RSV infection. Furthermore, exogenous expression of FBL in FBL-knockdown cells restored RSV infection and led to a concomitant reduction in ISG expression following the recovery of FBL protein levels. Finally, p53-knockdown cells reduced viral protein M2–1 levels without affecting FBL expression, pointing to the involvement of other regulatory mechanism controlling FBL during RSV infection.

**Conclusion:**

Our data show that RSV infection promotes the expression of the FBL protein, creating an environment devoid of early antiviral response mediators such as ISGS.

## 1 Introduction

Respiratory syncytial virus (RSV) is a negative-polarity RNA-enveloped virus that belongs to the *Pneumoviridae* family. Its genome is approximately 15.2 kb and encodes 10 genes that give rise to 11 proteins, of which nine are structural, and two are nonstructural. RSV is a highly contagious pathogen that primary affects the lower respiratory tract and can be potentially fatal in neonates, older adults, and immunocompromised people. Globally, RSV represent a major concern due to the weak protective immunity generated following infection (Kaler et al., 2023; Ciapponi et al., 2024). Neutralizing antibodies generated during primary infection provide only partial protection, and low activation of T cells is observed during reinfection (Bont et al., 2002). This explains why both children and adults can experience multiple reinfections throughout life (Georgakopoulou and Pitiriga, 2025). RSV has a tropism mainly for the ciliated epithelial cells of the respiratory tract; however, it has also been reported to infect cells in other organs and tissues, including the central nervous system (CNS) and placenta (Velázquez-Cervantes et al., 2019; Pollard et al., 2024). In addition, viruses can mutate, further complicating the development of effective vaccines and hinders the control of infections. In recent years, RSV vaccines for adults and a monoclonal antibody for prophylactic therapy for infants have been introduced. Nevertheless, these strategies fail to address critical needs, such as infant vaccination in low-income countries (Shaaban et al., 2025).Continues researching into RSV biology, its interaction with the immune system, and the development of more effective therapies and vaccines remains essential (Topalidou et al., 2023).

The nucleus is a highly specialized cellular compartment that houses the genetic information necessary for the regulation of cellular functions. Precise control of gene expression relies on tightly regulated mechanisms that modulate the transport of proteins, mRNA, and protein-RNA complexes between the nucleus and the cytoplasm (Lafontaine et al., 2021). Notably, many viruses have evolved strategies to hijack manipulate this trafficking. For example, poliovirus and severe acute respiratory syndrome coronavirus (SARS-CoV) inhibit the nuclear import of the host proteins, whereas viruses such as influenza A virus interfere with the nuclear export of host mRNA (Gustin and Sarnow, 2001; Satterly et al., 2007; Gomez et al., 2019). The nucleolus is normally recognized as a primary site of ribosomal RNA (rRNA) biogenesis, where rRNA are transcribed, processed, and assembled into ribosomal subunit. Theses subunits are subsequently exported to the cytoplasm to participate in proteins synthesis. However, accumulating evidences over recent years have revealed that the nucleolus also plays broader roles in diverse cellular processes, including cell cycle regulation, apoptosis, and the DNA damage response (Sakthivel et al., 2023). In addition, emerging studies suggest that the nucleolus contributes to the regulation of immune response and inflammatory signaling (Lee et al., 2022).

Fibrillarin (FBL) is a core nucleolar protein with a well-established role in ribosome biogenesis and nucleolar organization. It is a 34 kDa S-adenosylmethionine–dependent methyltransferase that is highly conserved across Archaea and Eukarya and catalyzes the site-specific methylation of ribosomal RNAs. Structurally, FBL is composed of four main domains: a spacer region, an RNA-binding domain, a methyltransferase domain, and an alpha-helical region. In eukaryotic cells, FBL functions as part of small nucleolar ribonucleoprotein (snoRNP) complexes, where it associates with proteins such as Nop56 and Nop58, as well as with small nucleolar RNAs (snoRNAs), to regulate rRNA processing and modification (Zhang et al., 2024). Together, these proteins and RNAs form ribonucleoprotein complexes. FBL is specifically found in the nucleoli and cajal bodies, it is commonly used as a nucleolus marker. FBL, also functions as atranscriptional regulator, cellular stress sensor, and ribonuclease (Zhang et al., 2024). Recent evidence suggests that FBL also contributes to immune regulation. Tiku and collaborators reported that siRNA-mediated knockdown of FBL reduced colony-forming units (CFUs) of *Staphylococcus aureus* (*S. aureus*) in murine bone marrow-derived macrophages. They also observed that cells lacking FBL survived longer following *S.aureus* infection compared with cells expressing FBL. This effect was attributed to decreased secretion of pro-inflammatory cytokines and increased production of anti-inflammatory cytokines (Tiku et al., 2018). In the case of viral infections, the vesicular stomatitis virus (VSV) Li et al. found that FBL inhibition affects early stages of VSV infection cycle. To evaluate the mechanism by which FBL affected the infection cycle, Li et al. performed RNA-seq, which revealed that the absence of FBL increased the expression of antiviral genes (Li et al., 2022). On the other hand, Deffrasnes et al. demonstrated that FBL is important during the early stages of Hendra virus (HeV) infection, as the clearance of FBL in HeV-infected HeLa cells caused a reduction in the production of infectious viral particles. This effect was attributed to the catalytic methyltransferase activity of FBL, which is necessary for infection, since cells lacking catalytically active FBL had a reduction in the production of viral particles. However, it remains unclear whether fibrillarin regulates viral infection through nucleolar functions, relocation during infection, or modulation of host transcriptional responses (Deffrasnes et al., 2016).

It has been reported that several negative-strand RNA viruses can traffic viral proteins to the host cell nucleus. For example, the matrix (M) protein of viruses such as Hendra virus (HeV), Nipah virus, and Newcastle disease virus localizes to the nucleus during the early stages of infection, where it can promote processes such as transcriptional regulation through direct interactions with host chromatin (Duan et al., 2014; Rawlinson et al., 2018; Zhao et al., 2025). Similarly, the RSV nonstructural protein NS1 has been shown to bind chromatin regulatory elements to modulate host gene expression (Pei et al., 2021). Notably, the matrix protein of HeV has been reported to interact with the nucleolar protein fibrillarin (FBL), thereby influencing mRNA expression (Deffrasnes et al., 2016). While the RSV M protein and M protein form other paramyxoviruses are primarily involved in viral assembly and egress (Förster et al., 2015; Liu et al., 2018). In this study, we aimed to characterize the role of fibrillarin (FBL) in regulating host innate antiviral transcriptional responses during RSV infection and to assess the consequences of FBL modulation for viral cycle replication.

## 2 Materials and methods

### 2.1 Molecular docking analysis

To investigate whether FBL might interact with the RSV matrix protein, an *in silico* analysis was performed. Given that the matrix protein of Hendra virus has been reported to interact with FBL, this interaction was used as a reference for the bioinformatic analysis. Firstly, the amino acid sequences of the RSV Matrix protein (ID: 1494472) and the Hendra virus matrix protein (ID: 1446472) were collected in the NCBI database. Sequence alignments were then performed using the UniProt Aling server (https://www.uniprot.org/align) to determine the degree of homology between the proteins. Following this analysis, the Protein Data Bank (PDB, www.rcsb.org) was queried to identify available crystallographic structures of the fibrillarin (2IPX), the RSV matrix protein (4V23), and Hendra virus matrix protein (6BK6) enabling, subsequent molecular docking studies. The obtained PDBs structures were preprocessed to remove non-biological elements introduced during X-ray crystallography, including water molecules, ligands, and crystallization agents. The cleaned structures were then submitted to the GalaxyWEB (https://galaxy.seoklab.org/) server for structural refinement, generating optimized three-dimensional models with improved stereochemical and energetic properties. Molecular docking analysis were subsequent performed using the Cluspro server (https://cluspro.bu.edu/), and the docked complex with the lowest interaction energy was selected for further analysis. Finally, to obtain insights into potential interaction regions, the CABS-FLEX (http://biocomp.chem.uw.edu.pl/CABSflex2) server was used to analyze the flexible regions of the RSV and Hendra virus matrix proteins. Following the same workflow, an additional *in silico* analysis was performed to evaluate a potential interaction between FBL and the RSV NS1 protein (ID: 5VJ2), using the same tools and parameters applied in the analysis of the interaction between FBL and the matrix protein.

### 2.2 Cell culture, viral strain, and reagents

The RSV A Long (ATCC, VR-26) viral stock was generated by propagation in Hep-2 cells. Cell cultures were maintained in Dulbecco’s Modified Eagle Medium (DMEM; Gibco, USA) supplemented with 5% fetal bovine serum (Gibco, USA) and antibiotic (penicillin 5 × 104 U/mL and strepto-mycin 50 μg/mL; Gibco, USA) at 37 °C and humidified atmosphere containing 5% CO_2_. Additionally, viral titers were determined by a plaque forming unit (PFU) assay (McKimm-Breschkin, 2004). Briefly, tenfold serial dilution of virus stock was prepared in DMEM and used to infect confluent Hep-2 cells monolayers in 24-well plates. After incubation at 37 °C for 2 h, the inoculum was removed and cells were overlaid with a DMEM medium containing 3% methylcellulose (Sigma- Aldrich, USA). Cultures were then incubated for 5 days under the same conditions, and plaques were visualized by staining with napthol-blue black solution to quantify PFUs for subsequent experiments.

Epithelial cell line A549 (ATCC CCL-185™) was grown in Advanced F12K Medium (Gibco, USA) supplemented with 2 mM glutamine, antibiotics (penicillin 5 × 104 U/mL and streptomycin 50 μg/mL; Gibco), 10% fetal bovine serum (Gibco) and 10 mL/L pyruvate (Gibco, USA) at 37 °C and 5% CO_2_ atmosphere.

### 2.3 RNA interference assay

A total of 4 × 10^5^ A549 cells were transfected with small interfering RNA (siRNA) targeting FBL (Thermo Fisher Scientific #s4820), siRNA targeting p53 (Santa Cruz Biotechnology, #sc-29435), or a scrambled siRNA control (Thermo Fisher Scientific #4390843) using Lipofectamine™ 2000 reagent, according to the manufacturer’s instructions. Transfections were performed at siRNA concentrations of 10, 20, and 30 μM and incubated for 12, 18, 24, 36, and 48 h. The efficiency of FBL silencing was assessed by Western blot analysis.

### 2.4 FBL and P53 silencing and RSV infection in A549 cells

A549 cells were subjected to gene silencing using siRNA targeting FBL, siRNA targeting p53 and scrambled siRNA control, under the conditions described above. 24 h after transfection, cells were infected with the virus at a multiplicity of infection (MOI) of 1 MOI. Subsequent assays were performed 24 hours post-infection to evaluate the effects of silencing.

### 2.5 Immunofluorescence assay

RSV-infected and siRNA-treated A549 cells were fixed with 4% paraformaldehyde (Sigma-Aldrich), permeabilized with PBS-Triton X-100 (0.1%) and gelatin (0.05%), and blocked with PBS containing 10% fetal bovine serum (FBS). The following primary antibodies were used: mouse anti-RSV nucleoprotein (1:100, 1 h; Genetex, USA, GTX636710), rat anti-RSV matrix protein (1:100, overnight; generated in-house), and rabbit anti-FBL (1:100, 1 h; Abcam, United Kingdom, ab226178). After three washes with PBS-Triton X-100 (0.1%), the appropriate secondary antibodies were applied: FITC-conjugated anti-mouse (1:300; Thermo Scientific #31547), anti-mouse Cy3 (1:200; Thermo Scientific, c2343), and anti-rabbit Cy3 (1:200; Thermo Scientific, A-11008). Images were acquired using a Zeiss LSM 900 confocal microscope. Random fields were analyzed to quantify the percent of infected cells and the number of inclusion bodies per cell, and these data were used to generate corresponding graphs.

### 2.6 Western blot assay

A total of 4x10^5^ A549 cells were cultured in 12-well plates. A total of 4 × 10^5^ A549 cells were cultured in 12-well plates. Cells were transfected for 24 h and subsequently infected at a multiplicity of infection (MOI) of 1 for an additional 24 h. After infection, cells were lysed and proteins were extracted using M-PER reagent (Thermo Fisher, #78501) supplemented with 1× protease inhibitor cocktail (Thermo Fisher, #87786). Protein concentration was determined by the Bradford assay using bovine serum albumin (Sigma-Aldrich) as a standard and dye reagent concentrate (Bio-Rad, USA). Western blot analysis was performed under denaturing conditions using a 10% SDS–polyacrylamide gels, followed by protein transfer onto nitrocellulose (Santa Cruz, USA) according to standard protocols. Membranes were incubated with primary antibodies against RSV M2-1 (mouse, 1:500, 1h; Abcam, United Kingdom, ab94805), GAPDH (mouse, 1:2000, 1 h; Santa Cruz Biotechnology, USA, sc-32233), and FBL (rabbit, 1:250, 1 h; Santa Cruz Biotechnology, sc-166001). After incubation with the appropriate HRP-conjugated secondary antibodies (anti-mouse or anti-rabbit IgG, 1:3000, 1 h Thermo Fisher Scientific, 31460) signal were detected using a chemiluminescent substrate kit (SuperSignal™ West Femto, Thermo Scientific).Densitometric analysis of the bands was performed using ImageJ software (NIH, USA); to quantify relative protein expression levels and to assess differences between experimental conditions.

### 2.7 siRNA treatment in A549 cells infected with RSV

The anti-RSV activity of FBL-targeting siRNA was evaluated in A549 cells seeded at a density of 1.2 × 10^5^ cells per well in 24-well plates for immunofluorescence assays, 4 × 10^5^ cells per well in 12-well plates for protein analysis, and 8 × 10^5^ cells per well in 6-well plates for RNA extraction. Cells were transfected with siRNA at a final concentration of 20 μM. Twenty-four hours after transfection, cells were infected with RSV at a multiplicity of infection (MOI) of 1 and incubated for 2 h to allow viral adsorption. The inoculum was then removed, cells were washed to eliminate unbound virus, and the antiviral effect was evaluated 24 h post-infection. The same experimental procedure was performed for p53 siRNA-treated cell.

### 2.8 RNA extraction and RT−qPCR

Total RNA was isolated from RSV-infected A549 cells treated with FBL siRNA and from RSV-infected cells treated with control siRNA using TRIzol reagent (Thermo Fisher Scientific, USA). RNA concentration and purity were determined using a NanoDrop spectrophotometer (Thermo Scientific, USA) and equal amounts of RNA were reverse transcribed into cDNA using a commercial reverse transcription master mix (RevertAid kit K1691, Thermo Scientific). Quantitative real-time PCR (RT-qPCR) was performed using SYBR™ Green Universal PCR Master Mix (Thermo Fisher Scientific). primer sequences for qPCR analysis are listed in **Supplementary Table 1.**

### 2.9 Impact of FBL inhibition on the production of infectious RSV particles

To analyze the effect on viral replication, A549 cells were treated with the FBL siRNA. After 24 hours of transfection, the cells were infected with RSV at an infection multiplicity (MOI) of 1. Twenty-four hours post-infection, supernatants were collected and evaluated by plaque-forming unit (PFU) assay. In parallel, twenty-four hours after siRNA transfection, cells were transfected with a plasmid encoding FBL to restore FBL expression. Cells were subsequently infected with RSV at a multiplicity of infection (MOI) of 1, and viral titers in culture supernatants were determined by plaque-forming unit (PFU) assay.

### 2.10 Construction of the FBL-GFP plasmid

The pEGFP-N1 plasmid, codon-optimized for expression in mammalian cells and encoding the fibrillarin (FBL) protein, was used as the cloning vector. Standard PCR techniques were employed to amplify the FBL insert using primers listed in **Supplementary Table 1.** Both the plasmid and the PCR-amplified insert were digested with the restriction enzymes *BglII* (New England Biolabs, #R0144S) and *HindIII* (New England Biolabs, #R0104S). Following digestion, the plasmid vector was dephosphorylated using alkaline phosphatase (Thermo Fisher Scientific, #EF0652).

Ligation was performed using T4 DNA ligase (Thermo Fisher Scientific, #EL0014), and the ligation products were used to transform *Escherichia coli DH5α* competent cells, along with the appropriate negative and positive controls. A single colony was selected and re-streaked, and plasmid DNA was purified using the GeneJET Miniprep Kit (Thermo Fisher Scientific, #K0503). Diagnostic restriction digestion with *BglII* and *HindIII* was subsequently performed to confirm the presence and expected size of the FBL insert.

After verification of the correct construct, a second bacterial transformation was carried out to obtain larger quantities of plasmid DNA, followed by large-scale plasmid purification using a QIAGEN Plasmid Maxi Kit.

### 2.11 Restoration of FBL expression via plasmid transfection

To analyze whether the inhibitory effect on the RSV infection cycle is mediated by FBL inhibition, a protein restoration approach was performed by transfecting cells with a plasmid containing the FBL sequence to allow its expression. In this case, A549 cells were initially transfected with FBL-targeting siRNA following the conditions described above. Twenty-four hours post-transfection, cells were transfected with the p-EGFP-N1 plasmid containing the FBL sequence, and 24 h later they were infected with RSV at a multiplicity of infection (MOI) of 1. Finally, 24 h post-infection, some cells were fixed for evaluation, while proteins and RNA were extracted from the remaining cells, following the procedures previously described.

### 2.12 FBL overexpression and RSV infection

To evaluate the effect of FBL overexpression on RSV infection, A549 cells were transfected with the p-EGFP-N1 plasmid containing the FBL sequence. After 24 h of transfection, cells were infected with RSV at a multiplicity of infection (MOI) of 1. At 24 h post-infection, cells were either fixed for further analysis or lysed for protein extraction using M-PER reagent (Thermo Fisher, #78501) supplemented with 1× protease inhibitor cocktail (Thermo Fisher, #87786).

### 2.13 Statistical analysis

Data are expressed as the mean ± standard error of the mean (SEM) from experiments performed in triplicate. Statistical analyses were conducted using GraphPad Prism software (version 10.0). Comparisons between two groups were performed using an unpaired two-tailed Student’s *t*-test. For experiments involving more than two groups, one-way analysis of variance (ANOVA) was applied, followed by Dunnett’s *post hoc* test to compare each experimental group with the control. A *p* value ≤ 0.05 was considered statistically significant.

## 3 Results

### 3.1 RSV infection increased FBL expression in A549 cells

**Figure 1:**
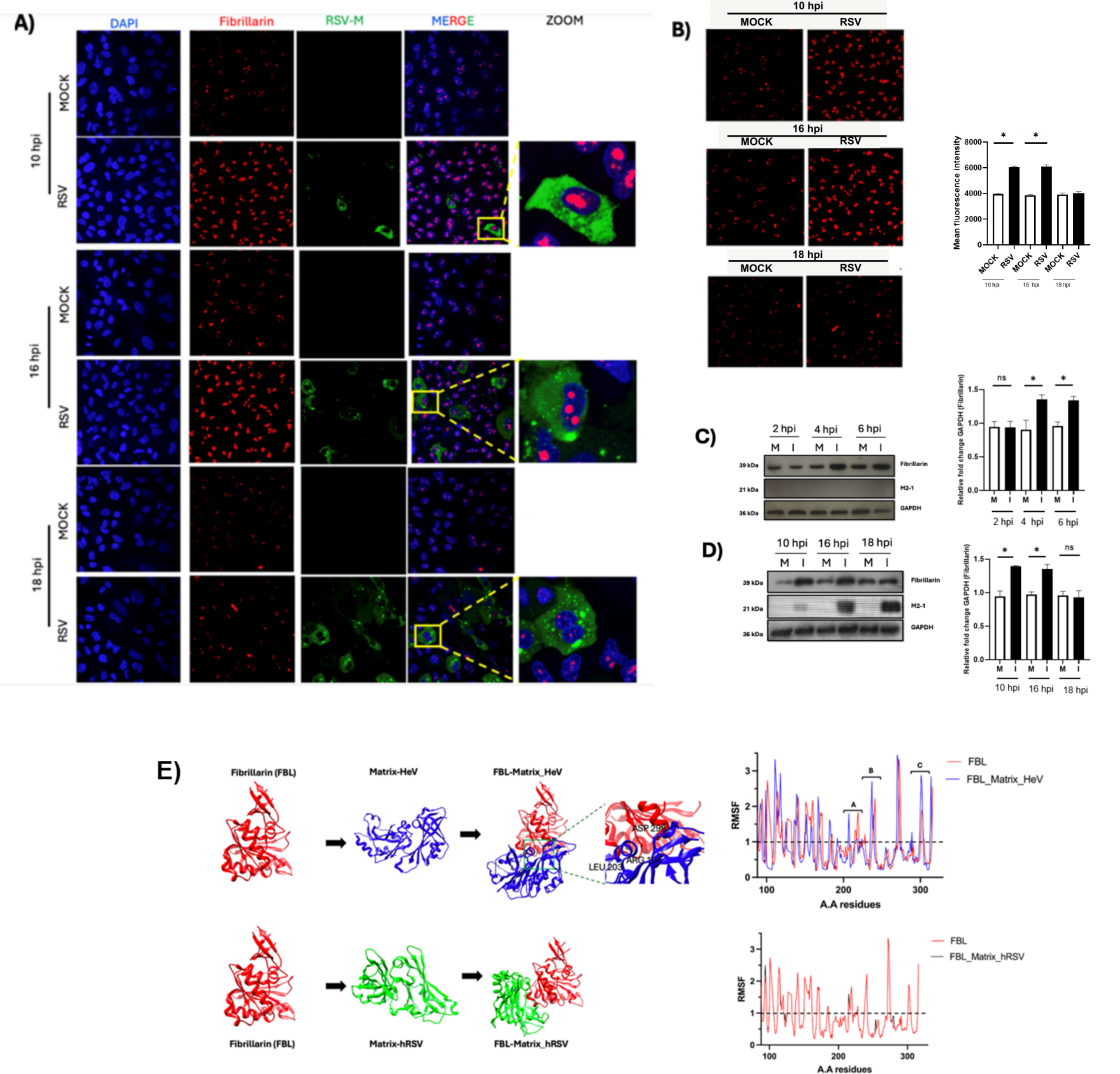
RSV promotes FBL increase in early times of infection **(A)** A549 cells infected with RSV at 1MOI at 10 h, 16 h and 18 h. Infected cells and falsely infected cells (MOCKs) are shown. At times 10 and 16 hours of infection, we can observe an accumulation or greater intensity of the FBL protein compared to MOCK cells. **(B)** ZOOM the areas where the FBL protein was found and FBL signal medium intensity analysis at times 10 h, 16 h and 18 h post-infection. **(C)** y **(D)**) Lysates of RSV- infected A549 cells at 1 MOI that were analyzed by WB. It was observed that at the times 4 h, 6 h, 10 h and 16 h post-infection there is an increase in the FBL protein compared to the times 2 h and 18 h post-infection. The viral protein M2-1 is used as infection control and the GAPDH protein is a load control. **(E)** Three-dimensional results obtained from ClusPro and the corresponding flexibility analysis using CABS-Flex are shown. RMSF analysis of FBL alone (red) and in complex with Hendra M (blue) or RSV M (black) highlights flexible regions (peaks) and rigid regions (troughs), indicating candidate interaction sites.P values are determined by unpaired two-tailed ttest. *P < 0.05. All data are representative of three independent experiments and are presented as means ± s.d.

Previous studies in members of the *Paramyxoviridae* family have shown that matrix (M) proteins can localize to the nucleus and, in some cases, associate with nucleolar components such as fibrillarin (FBL), suggesting conserved functions beyond viral assembly. In light of these observations, immunofluorescence assays were conducted at different times post-infection to examine the subcellular distribution of the RSV M protein and FBL. The analysis revealed that, at the time points evaluated, the nuclear localization patterns of the RSV M protein and FBL did not overlap (**Figure 1A**). Notably, RSV infection induced a marked redistribution and accumulation of the FBL fluorescent signal within infected cells. In mock cells, FBL exhibit a typical staining patter characterized by the presence of small dots structures whereas in RSV-infected cells an increase in the size dots, indicating altered nucleolar organization (**Figure 1B**). This accumulation was particularly evident at 10- and 16-hours post-infection, after which FBL localization progressively returned to a pattern comparable to that observed in mock cells by 18 hours post-infection (**Figure 1B**). The corresponding graph presents the quantified values along with statistical analysis demonstrating this difference.

To determinate whether this redistribution was associated by changes in FBL expression levels, total protein extracts were obtained from RSV-infected and control cells at different post-infection times. Western blot analysis showed that RSV infection induced an increase in FBL protein levels from early stages (4 hours post-infection), an effect that persisted until 16 hours and returned to levels comparable to MOCK conditions by 18 hours post-infection (**Figures 1C, D**). Densitometric quantification and statistical analysis confirmed the significance of these changes. Taken together, these results indicate that although no colocalization between the RSV M protein and FBL was detected under the conditions evaluated, RSV infection modulates both the expression level and nucleolar accumulation of FBL during the early stage of the viral replication cycle.

To further support the absence of colocalization observed between the RSV M protein and the FBL, an additional *in silico* analysis was performed to explore the theoretical feasibility of a direct interaction between these proteins. In section 1E, the interaction between the Henipavirus M protein and FBL is presented as a positive control, as this interaction has been previously reported. Subsequently, the potential interaction between the RSV M protein and FBL was evaluated. Docking models generated using ClusPro, together with flexibility analyses performed with CABS-Flex, showed that interaction with the Hendra virus M protein induced marked changes in FBL flexibility, consistent with a potential interaction. In contrast, docking with the RSV M protein did not induce significant alterations in FBL flexibility, supporting a negligible or weak interaction. Furthermore, flexibility analysis of NS1 in complex with FBL revealed localized changes in conformational dynamics, suggesting a potential interaction. Collectively, these computational analyses are consistent with the immunofluorescence results, reinforcing the conclusion that RSV M protein does not establish a stable or functionally relevant interaction with FBL under the conditions evaluated.

### 3.2 FBL silencing affects viral protein expression, inclusion body formation, and RSV replication

**Figure 2:**
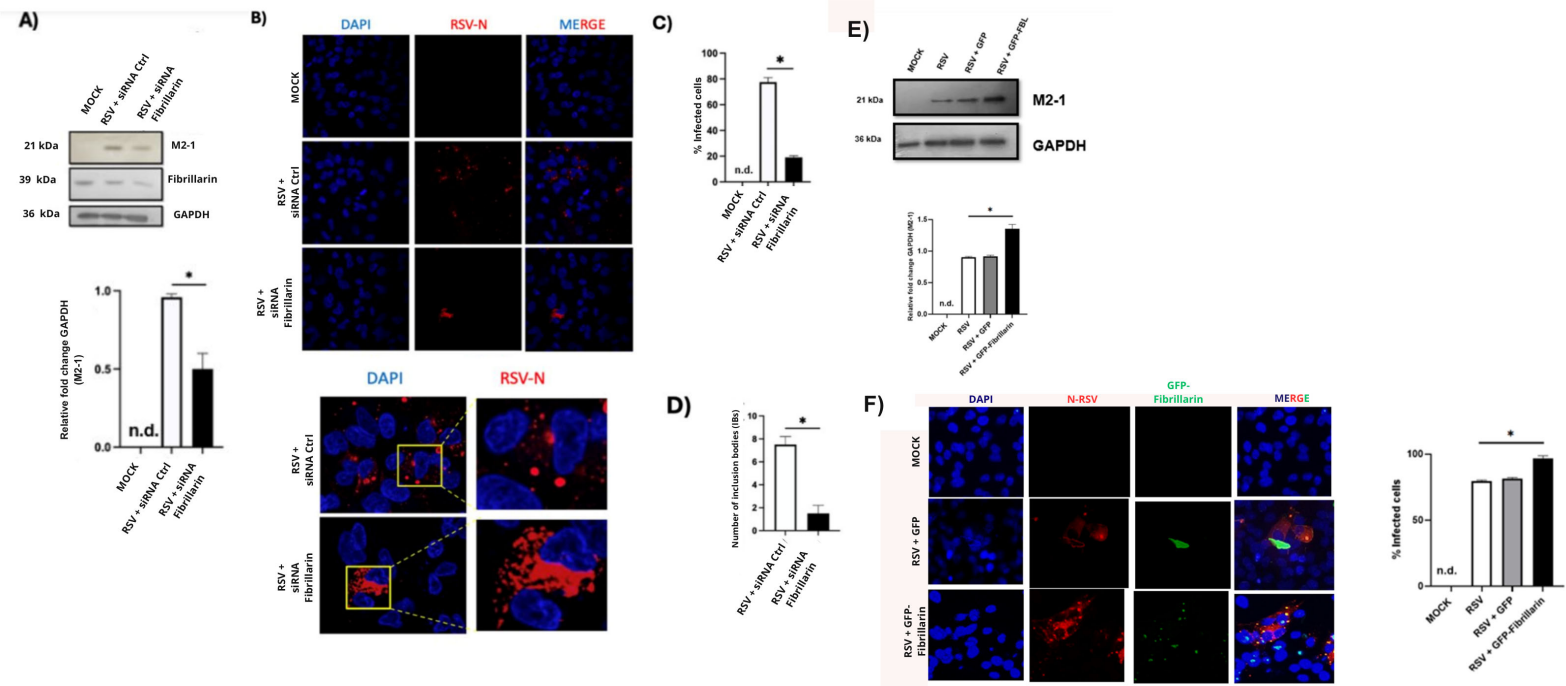
FBL silencing decreases infection. **(A)** A549 cells transfected with siRNA FBL and Ctrl. 24H post transfection was infected at 1MOI and 24 h post infection the cell protein was extracted. We observed that in the cells treated with siRNA FBL there is a decrease of the protein M2-1. **(B)** Immunofluorescence in which we observe that after transfection with siRNA FBL there is a reduction in the percentage of infected cells. **(C)** Analysis of figure B). **(D)** Analysis of number of inclusion bodies (IBs) present in figure B). n.d (not detected). **(E)** A549 cells transfected with GFP- FBL and Ctrl. 24H post transfection was infected at 1MOI and 24 h post infection with the cell protein was extracted. We observed that in the cells treated with GFP- FBL there is a increase of the protein M2-1. *P* values are determined by unpaired two-tailed *t*test. *P < 0.05. All data are representative of three independent experiments (n= 10 fields) and are presented as means ± s.d.

**Figure 3:**
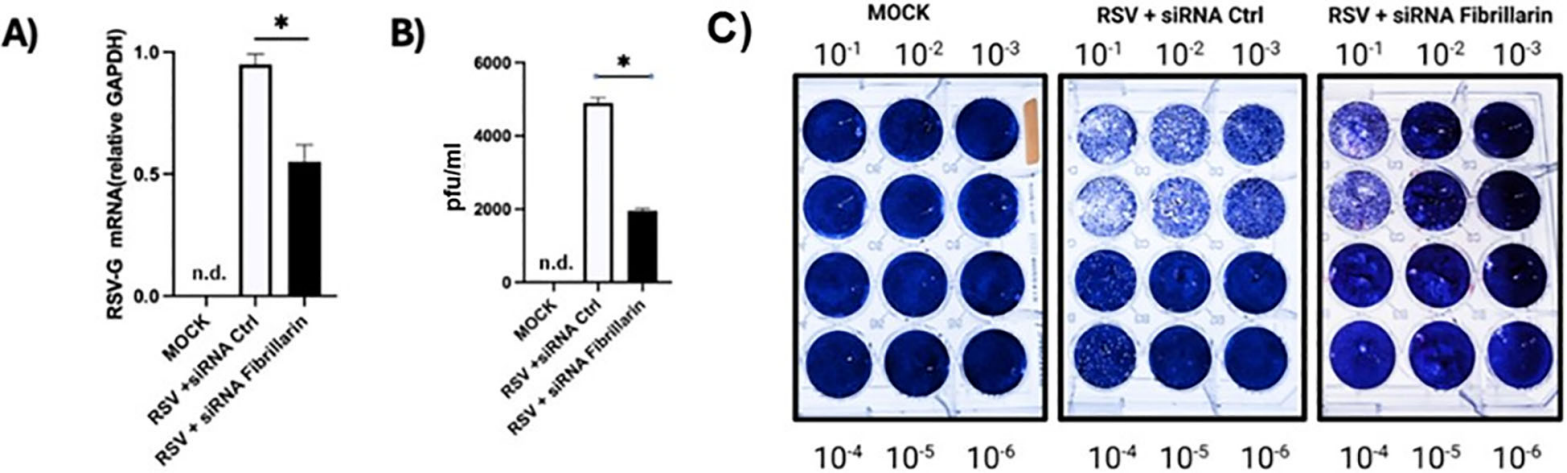
FBL silencing reduces RSV replication and the production of infectious viral particles. **(A)** Analysis of the relative mRNA expression of the RSV F gene, showing a significant decrease in G-RSV mRNA levels in A549 cells treated with F-specific siRNA. **(B)** Plaque assay performed in RSV-infected A549 cells treatment with FBL siRNA, demonstrating a reduction in the production of infection viral particle compared with control cells. **(C)** Representative images of the PFUs assay under Mock, RSV + siRNA Ctrl, and RSV + siRNA Fibrillarin. n.d (not detected). Statistical significance was determined using an unpaired two-tailed Student’s t-test- P<0.05. Data are representative of three independent experiments and are presented as mean ± standard deviation (SD).

Given the potential role of fibrillarin (FBL) in RSV infection, the impact of its partial silencing on different stages of the viral replication cycle was evaluated. Western blot analysis revealed that inhibition of FBL expression led to a significant decrease in the levels of the RSV M2–1 protein, a key factor involved in viral transcription, as confirmed by densitometric analysis (**Figure 2A**). To determine whether this reduction was associated with alterations in viral transcription and replication sites, the formation of viral inclusion bodies was examined by immunofluorescence assays targeting the RSV nucleoprotein (N). In RSV-infected cells and no knockout FBL cells, the N protein exhibited a punctate cytoplasmic distribution, consistent with the presence of viral inclusion bodies as previously reported. In contrast, FBL-silenced cells displayed a more diffuse cytoplasmic distribution of the N protein, accompanied by a marked reduction in inclusion bodies formation (**figure 2B**).Under the experimental conditions described above, FBL silencing resulted in a significant reduction in the number of RSV-infected cells compared with cells treated with a non-targeting control siRNA (**Figure 2C**), suggesting that FBL contributes to efficient viral spread. Quantitative analysis revealed a significant decrease in the number of inclusion bodies under FBL silencing condition (**Figure 2D**). Consistent with these observations, Finally, to evaluate the functional consequences of FBL silencing in RSV-infected cells, viral RNA synthesis and the production of viral progeny were assessed. The results revealed that in RSV-infected cells subjected to FBL knockdown, the number of viral RNA copies corresponding to the G gene was reduced by approximately 50% compared with infected cells treated with a control siRNA (**Figure 3A**). In addition, a significant reduction in viral progeny production was observed in RSV-infected cells lacking FBL expression (**Figure 3B**), this effect is illustrated by representative PFU assay image obtained from supernatants of RSV-infected FBL knockdown cells compared with the controls (**Figure 3C**). Taken together, these results indicate that the RSV M protein does not appear to forms a functionally relevant complex with FBL. However, reduction of FBL expression limits the number of infected cells, disrupts the formation of viral inclusion bodies, impairs viral transcription and genome replication, and ultimately inhibits the production of infectious viral particles. These findings support the role of FBL as an essential host factor that facilitates RSV replication and spread. One of the questions we posed after observing that the partial absence of fibrillin leads to a decrease in infection was whether fibrillin overexpression would lead to an increase in infection. To investigate this, we transfected A549 cells with a plasmid expressing FBL and then infected them. What we observed (**Figures 2E, F**) is that fibrillin overexpression somewhat improved infection. It is important to mention that the phenomenon of increased fibrillarin expression during early RSV infection is possibly conserved, since in the Hep2 cell line we observed an increase in fibrillarin expression starting with 6 hours post-infection.

### 3.3 FBL silencing potentiates ISG expression in RSV-infected cells

Increasing evidence indicates that the nucleolus plays roles beyond ribosome biogenesis, including the regulation, processing, and turnover of specific pre-mRNAs associated with the innate immune response. In this context, nucleolar proteins have been implicated in modulating antiviral signaling pathways by influencing the expression of interferon-stimulated genes (ISGs), which are critical mediators of host defense against viral infections.

Given that ISGs constitute a central component of the antiviral response and considering our previous observations that FBL silencing markedly reduced RSV infection in A549 cells, we hypothesized that FBL may contribute to RSV replication by negatively regulating the expression of selected ISGs. To test this hypothesis, we quantified the mRNA levels of several key ISGs involved in antiviral signaling and viral RNA sensing, including *OAS1*, *OAS2*, *IFIT3*, *PKR*, *DDX58*, and *RIG-I*, under conditions of FBL silencing. Gene expression was normalized to both GAPDH and HPRT (**Supplementary Figure 2**). Comparable results were obtained with both reference genes, confirming the reproducibility of the transcriptional changes observed during infection.

**Figure 4:**
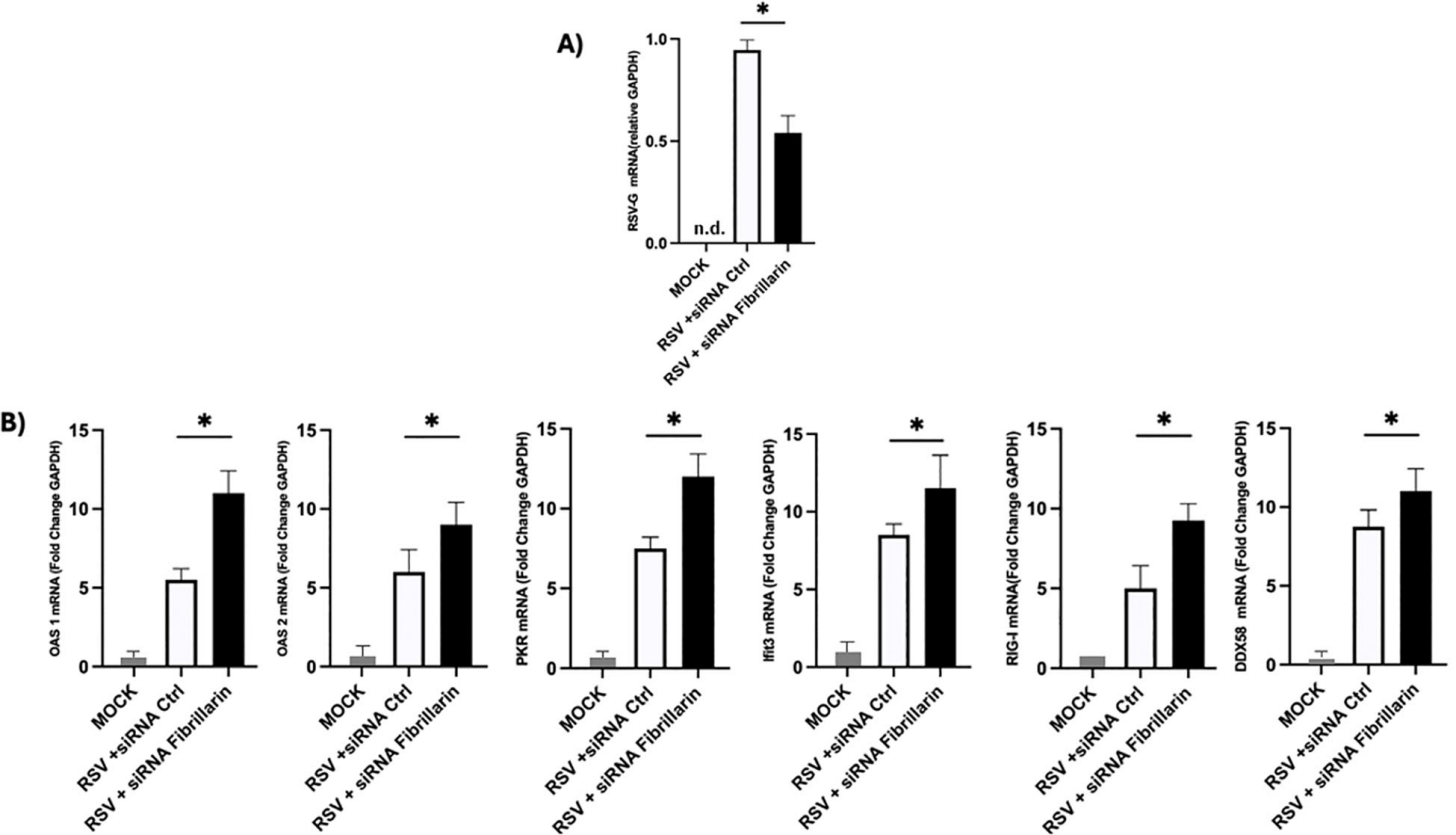
Silencing of the FBL increases the expression of ISGS. **(A)** A549 cells were transfected with FBL-specific siRNA and subsequently infected with RSV at a 1 MOI. FBL knockdown resulted in reduced expression of the G gene expression. **(B)** the silencing FBL expression was accompanied by significant increase in the expression of the ISGs: ISGs Oas1, Oas2, Ifiti3, PKR, DDX58 and RIG-I. n.d (not detected). Statistical significance was determined using a unpaired two-tailed Student’s t-test- *P<0.05. Data are representative of three independent experiment and are presented as mean ± standard deviation (SD).

As shown in **Figure 4**, inhibition of FBL expression resulted in a significant upregulation of all ISGs analyzed compared with control cells. This enhanced ISG expression suggests that the reduced RSV infection observed following FBL silencing may be mediated, at least in part, by the activation of a stronger antiviral state driven by ISG induction. Collectively, these findings support a model in which FBL acts as a negative regulator of innate immune gene expression, thereby facilitating RSV replication by dampening host antiviral responses. Interestingly, this same phenomenon is also found in the Hep2 cell line. **Supplementary Figure 2**.

### 3.4 Restoration of fibrillarin rescues RSV replication and viral protein expression in A549 cells

**Figure 5:**
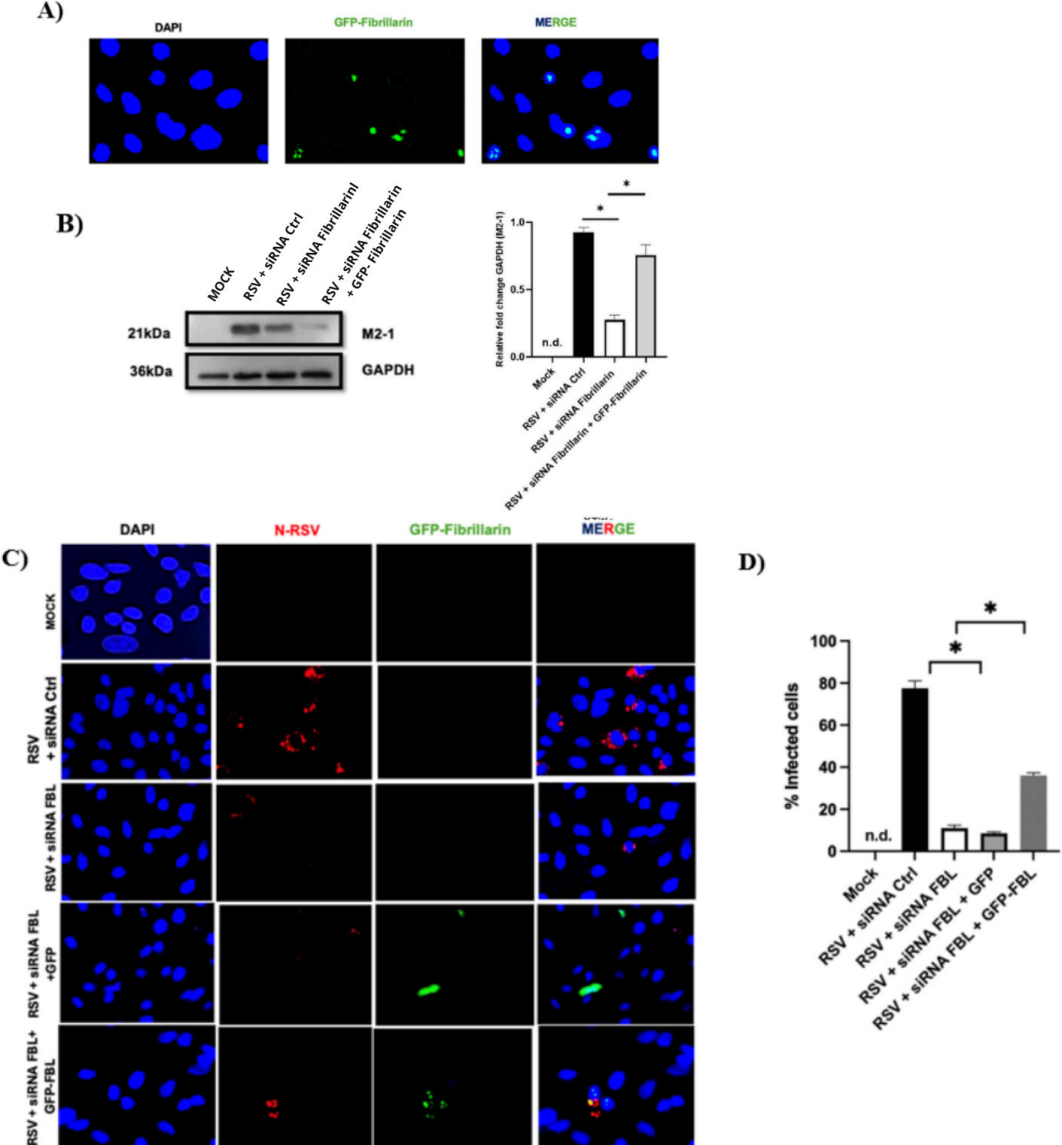
Fibrillarin Restoration Promotes RSV Replication and Viral Protein Expression in A549 Cells. **(A)** Exogenous fibrillarin expression in cells transfected with a fibrillarin-encoding plasmid. 24 hours post transfection. **(B)** RSV M2–1 protein levels in infected cells treated with control siRNA, FBL-targeting siRNA, or FBL siRNA followed by fibrillarin plasmid transfection. **(C)** Immunofluorescence showing RSV nucleoprotein (red) and fibrillarin (green) and nucleus (Blue). **(D)** Quantification of the percentage of RSV-infected cells under each condition. n.d (not detected). *P* values are determined by unpaired two-tailed *t*test. *P < 0.05. All data are representative of three independent experiments and are presented as means ± s.d.

To determine whether the effects of FBL silencing on RSV infection and interferon-stimulated gene (ISG) expression were directly attributable to FBL loss, we restored FBL expression in FBL-deficient cells using a GFP-FBL plasmid and evaluated the recovery of viral infection and ISG regulation. As shown in **Figure 5A**, exogenous expression of GFP-FBL in A549 cells resulted in a subcellular distribution comparable to that of endogenous FBL, characterized by predominant nucleolar localization with well-defined punctate structures within the nucleolus, consistent with the typical fibrillarin staining pattern.

**Figure 6:**
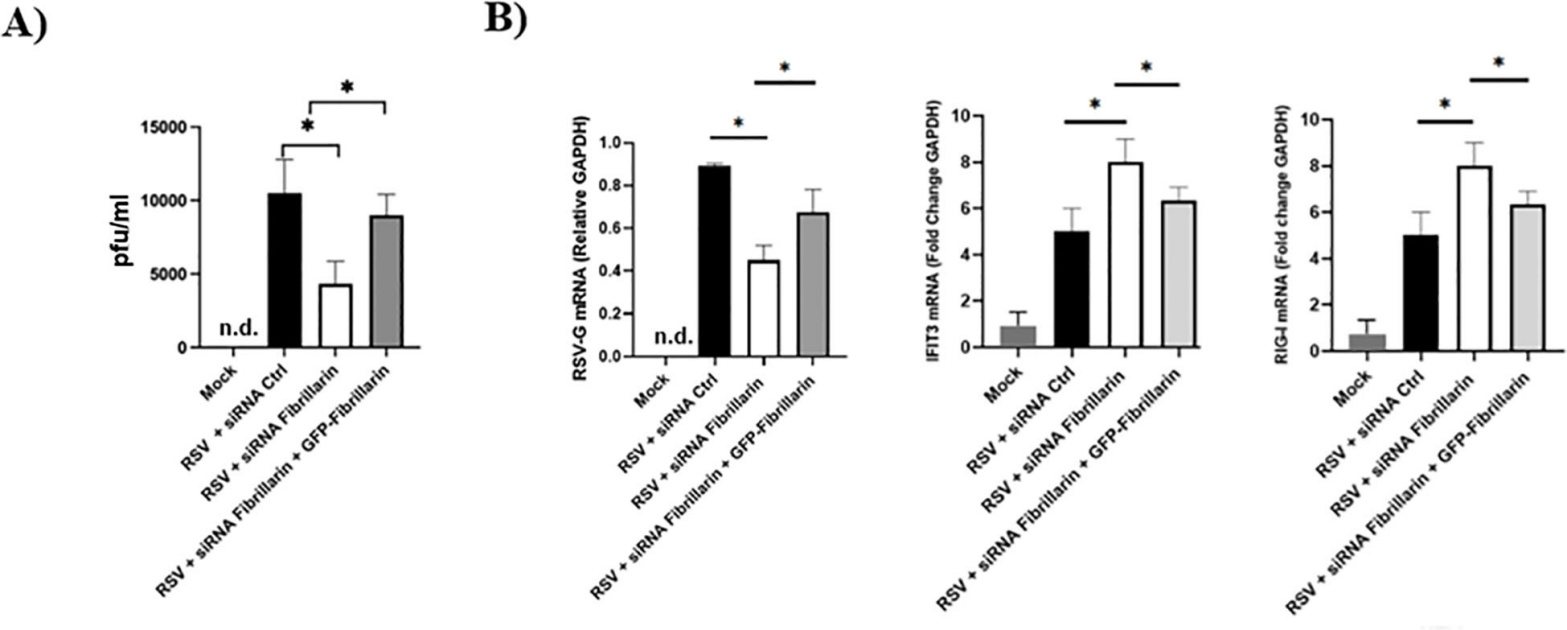
Effects of fibrillarin silencing and restoration on RSV infection and ISG expression. **(A)** Plaque assay of A549 cells infected with RSV and treated with control siRNA, FBL-targeting siRNA, or FBL siRNA followed by exogenous fibrillarin restoration. Restoration of fibrillarin increases the number of plaque-forming units (PFUs). **(B)** Analysis of ISG expression in A549 cells harvested 24 h post-transfection and 24 h post-infection under the same treatment conditions. Cells with fibrillarin restoration show a decrease in ISG levels compared with cells treated with FBL siRNA alone. n.d (not detected). P values were determined by unpaired two-tailed t-test. *P < 0.05. All data represent three independent experiments and are shown as means ± s.d.

**Figure 7:**
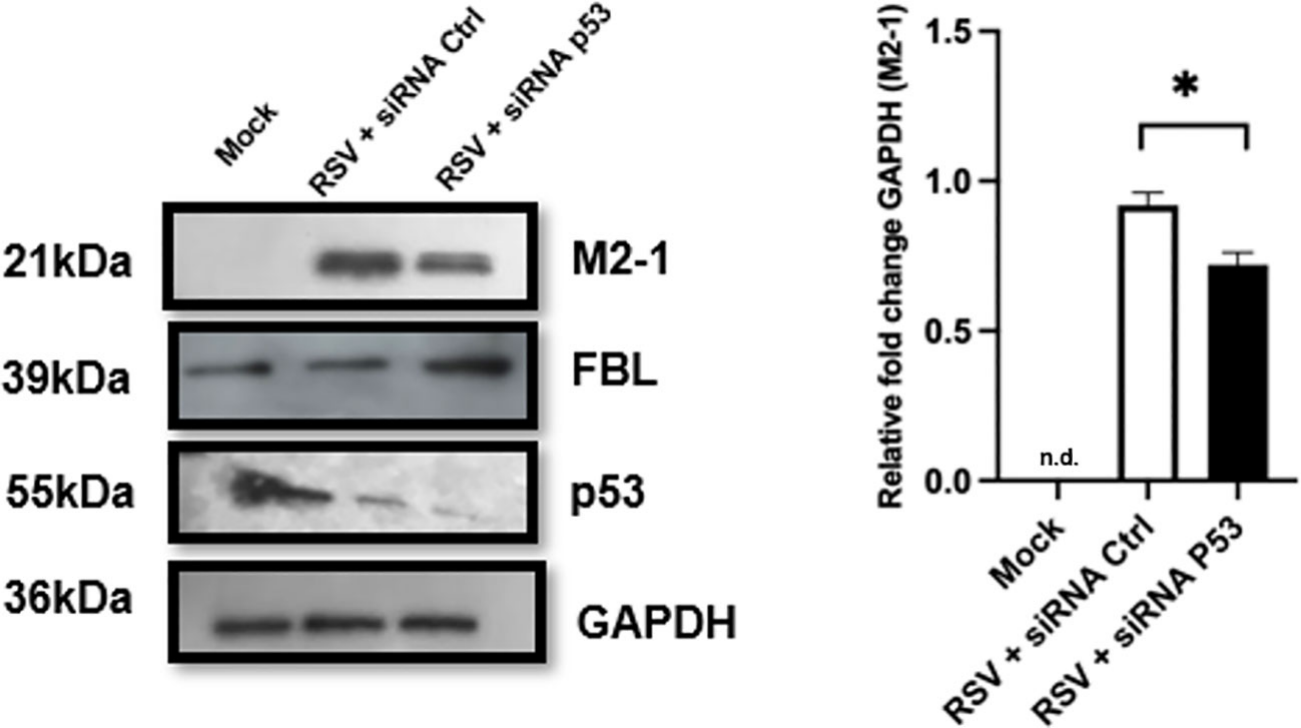
Regulation of fibrillarin (FBL) in RSV-infected A549 cells occurs partially independent of p53. RSV M2–1 and FBL protein levels were analyzed in A549 cells under different conditions: mock-infected cells, RSV-infected cells treated with control siRNA, and RSV-infected cells treated with p53-targeting siRNA, which resulted in an increase in FBL level. n.d (not detected). *P* values are determined by unpaired two-tailed *t*test. *P < 0.05. All data are representative of three independent experiments and are presented as means ± s.d.

Restoration of FBL expression in RSV-infected A549 cells subjected to endogenous FBL silencing led to a recovery of viral protein expression. Specifically, Western blot analysis revealed that re-expression of FBL restored the levels of the RSV M2–1 protein, which had been reduced upon FBL silencing (**Figure 5B**). Consistently, immunofluorescence analysis demonstrated that FBL restoration also increased RSV nucleoprotein (N) expression and promoted the reappearance of viral inclusion bodies, observed as spherical cytoplasmic structures, to levels comparable to those detected in RSV-infected cells treated with control siRNA notably, when cells were infected and transfected with the parental plasmid, the distribution and infection pattern observed by immunofluorescence were like those in cells treated with control siRNA (**Figure 5C**). In agreement with these observations, plaque formation assays showed that restoration of FBL partially rescued viral progeny production. Analysis of supernatants from cells with restored FBL expression revealed a higher number of infectious viral particles compared with FBL-silenced cells, although these levels did not fully reach those observed in RSV-infected cells treated with control siRNA (**Figure 6A**). Similarly, quantification of viral RNA indicated that FBL restoration promoted a partial recovery of viral replication (**Figure 6B**). Finally, to assess whether FBL restoration also affected the antiviral response, the expression levels of interferon-stimulated genes (ISGs) were measured in RSV-infected A549 cells subjected to FBL silencing followed by FBL re-expression. As shown in **Figure 7B**, restoration of FBL resulted in a reduction of ISG expression levels, reversing, at least in part, the ISG upregulation observed under FBL silencing conditions. Collectively, these results demonstrate that fibrillarin restoration partially rescues RSV infection by promoting viral protein expression, inclusion body formation, viral genome replication, and progeny production, while concomitantly dampening the antiviral ISG response. These findings further support a functional role for FBL as a host factor that facilitates RSV replication. Finally, we also decided to perform fibrillarin restoration in Hep2 cells partially lacking fibrillarin and observed the same phenomenon as in A549. All of this leads us to conclude that during RSV infection in A549 and Hep2 cells, fibrillarin is of vital importance for a correct infection cycle.

### 3.5 Fibrillarin regulation in RSV-infected A549 cells is partially independent of p53

The role of p53 during RSV infection has been extensively studied; however, reported findings have been inconsistent (Eckardt-Michel et al., 2008; Gladwell et al., 2023; Liu et al., 2024). While some studies have shown increased p53 levels during infection, others have reported a reduction, discrepancies that have been largely attributed to differences in experimental design, cell type, and RSV strain. Because p53 has been described as a regulator of FBL, we sought to evaluate the role of p53 during RSV infection, with particular emphasis on its function in regulating FBL expression. As shown in **Figure 7**. Western blot analysis was performed using protein extracts from RSV-infected cells treated with either control siRNA or p53-specific siRNA. In RSV-infected cells treated with control siRNA, low p53 levels were observed, consistent with previous reports by Liu and colleagues (Hiscox, 2007). In contrast, silencing of p53 in RSV-infected cells resulted in increased FBL protein levels, indicating a regulatory relationship between p53 and FBL during infection. Additionally, p53 knockdown led to a modest reduction in RSV infection compared with cells treated with control siRNA. Collectively, these findings indicate that p53 regulates FBL expression during RSV infection and suggest that modulation of the p53–FBL axis may influence viral replication dynamics.

## 4 Discussion

Although the nucleolus is classically recognized for its role in ribosomal RNA synthesis and ribosome assembly, it is now widely accepted as a multifunctional and dynamic nuclear compartment that integrates numerous cellular processes, including cell cycle regulation, DNA replication and repair, stress sensing, and transcriptional control (Muñoz-Velasco et al., 2025). Consistent with this multifunctionality, increasing evidence has implicated the nucleolus in the replication cycles of diverse viruses. During infection, particularly by negative-sense RNA viruses, viral proteins can traffic between the cytoplasm and nucleus, while nucleolar proteins may relocalize or participate in transient nucleolar remodeling, highlighting the importance of nucleo–cytoplasmic dynamics in viral replication and host manipulation (Hiscox, 2007).

Within the families *Paramyxoviridae* and *Pneumoviridae*, the matrix (M) protein is a highly conserved structural component. Beyond its role in viral assembly and budding, M proteins from several paramyxoviruses can transiently localize to the nucleus and interact with host factors, suggesting regulatory roles during infection (Förster et al., 2015; Liu et al., 2018). In related viruses such as Nipah and Hendra viruses, infection has been shown to alter the localization or expression of nucleolar proteins, including nucleophosmin, treacle, and fibrillarin (FBL), Based on these observations, we investigated whether RSV infection alters FBL dynamics and whether such changes could influence antiviral responses.

Immunofluorescence analyses revealed no colocalization between RSV M protein and FBL in the nucleolus of infected A549 cells. Consistent with this observation, in silico molecular docking analyses did not support a stable interaction between these proteins, suggesting that a direct association between RSV M and FBL in unlikely. This contrast with reports describing interactions between FBL and viral proteins from other RNA viruses, including PRRSV, mouse hepatitis virus, influenza A virus, HIV, and Hendra virus (Decle-Carrasco et al., 2023). Despite functional similarities between the M proteins of RSV and Hendra virus in viral assembly and nuclear trafficking, substantial differences in their amino acid sequences and structural organization likely account for their distinct interaction profiles with nucleolar host factors. Structural features may further explain these differences. Although both proteins require oligomerization for viral assembly, RSV and Hendra virus M proteins rely on different residues for stable dimmer formation. In RSV, residues such as S63, N93, and Y229 contribute to dimer stability, whereas Hendra virus M dimerization involves residues including R118, T120, V154, R158, and E166 (Liu et al., 2018). Additionally, RSV M oligomerization can be regulated by phosphorylation at Thr205, a mechanism not reported for Hendra virus M. These structural distinctions may influence the capacity of each protein to interact with specific host partners (Duan et al., 2023).

Taken together, these observations provide a structural and mechanistic basis for the lack of nucleolar localization and interaction between the RSV matrix (M) protein and fibrillarin (FBL). Although RSV NS1 emerged as an alternative candidate based on *in silico* docking analyses suggesting a potential interaction with FBL, these results should be interpreted with caution. In the study reporting nuclear localization of NS1, this redistribution was observed at 24 hours post-infection, a time point later than those associated with early host responses. Moreover, in the same study, a proteomic analysis of proteins co-immunoprecipitated with NS1 failed to identify FBL as an interacting partner (Pei et al., 2021). Together, these findings indicate that, despite the docking predictions and the ability of NS1 to translocate to the nucleus at later stages of infection, there is currently no experimental evidence supporting a direct interaction between NS1 and FBL. Therefore, any potential NS1–FBL interaction would likely be transient, indirect, or restricted to specific stages of infection, and further experimental validation will be required to conclusively address this possibility.

Functional experiments demonstrated that FBL contributes to RSV infection. siRNA-mediated silencing of FBL significantly reduced the number of infected cells, viral genome replication, and production of infectious viral particles. In addition, depletion of FBL disrupted the formation of viral inclusion bodies, structures that serve as sites of viral replication and transcription. FBL knockdown resulted in fewer inclusion bodies and altered localization of the viral nucleoprotein (N), suggesting that FBL may facilitate the proper organization of these replication compartments. Given that inclusion bodies have been shown to sequester host cellular factors such as NF-κB, preventing nuclear translocation of the p65 subunit and limiting the activation of antiviral responses, disruption of their formation provides a mechanistic explanation for the reduced viral infection observed upon FBL depletion (Jobe et al., 2020). Consistent with these findings, overexpression of FBL promoted enhanced RSV infection, further supporting a proviral role for this nucleolar protein.

Recent studies have highlighted emerging roles for nucleolar proteins in innate immune regulation. Silencing of FBL has been associated with increased expression of antiviral genes, including interferon-stimulated genes (ISGs) such as OAS2, IFIT1, and IFIT2. In other viral systems, including vesicular stomatitis virus, FBL has been shown to regulate ISG expression through RNA methylation–dependent mechanisms that influence activation of the MDA5 antiviral sensing pathway (Li et al., 2022). These findings suggest that FBL may function not only in ribosome biogenesis but also in the regulation of antiviral signaling pathways. This mechanism could also explain the results observed in our study, where silencing of FBL reduced RSV infection, potentially as a consequence of enhanced antiviral responses in FBL-depleted cells. Another possible mechanism could involve regulation of immune transcripts through the Rrp6–exosome complex. Studies on nucleolin, another nucleolar protein, have shown that its depletion enhances immune gene expression by preventing the recruitment of transcripts to this degradation pathway (40). It will therefore be interesting in future studies to determine whether FBL plays a similar role during RSV infection, potentially modulating the expression of interferon-stimulated genes (ISGs).

Given that changes in FBL expression were detected as early as 4 hours post-infection, when viral proteins are not yet reported to localize to the nucleus, host cellular factors may contribute to the regulation of FBL during RSV infection. One possible candidate is the transcription factor p53, which has been shown to regulate FBL transcription and whose levels increase during early stages of RSV infection. However, in our model p53 silencing resulted in only a modest reduction in viral infection, suggesting that although p53 may participate in FBL regulation, it is unlikely to be the sole determinant. This is consistent with previous reports describing contrasting roles for p53 during RSV infection, with some studies indicating proviral functions that promote replication, while others report antiviral effects through modulation of inflammatory signaling. Together, these observations suggest that additional transcriptional factors or cellular signaling pathways may contribute to the modulation of FBL expression during RSV infection (Eckardt-Michel et al., 2008; Gladwell et al., 2023; Liu et al., 2024).

In conclusion, our findings demonstrate that RSV infection induces a transient increase in the expression of the nucleolar protein fibrillarin (FBL). This modulation appears to be functionally relevant for the viral cycle, as reduction of FBL expression results in a significant decrease in RSV infection. Our data further suggests that FBL may contribute to the regulation of interferon-stimulated gene expression, highlighting a previously underappreciated role for nucleolar proteins in shaping antiviral immune responses. Together, these findings provide new insight into host–virus interactions at the nucleolar level and identify FBL as a potential target for therapeutic strategies aimed at limiting RSV infection.

## Data Availability

The raw data supporting the conclusions of this article will be made available by the authors, without undue reservation.

## Supplementary material

Supplementary Figure 1 *In silico* analysis to determine the interaction of RSV Non-Structural 1 protein with FBL cell protein. **(A)** Molecular docking analysis showing the three-dimensional representation of the respiratory syncytial virus (RSV) non-structural protein 1 and the fibrillarin protein. **(B)** Flexibility analysis of fibrillarin alone and in complex with the RSV non-structural protein 1, assessed by root mean square fluctuation (RMSF) of amino acid residues. Fibrillarin alone is shown in red, whereas fibrillarin in complex with the RSV non-structural protein 1 is shown in black. Higher RMSF values indicate regions with increased flexibility, while lower values correspond to more rigid regions.

Supplementary Figure 2 RSV promotes FBL increase in early times of infection in HEP-2 cells. **(A)** Lysates of RSV- infected HEP-2 cells at 1 MOI that were analyzed by WB. It was observed that at the times 6 h, 10 h and 16 h and 18 h post-infection there is an increase in the FBL protein compared to the times 2 h and 4h h post-infection. **(B)** HEP-2 cells transfected with siRNA FBL e infected with RSV at 1 MOI, where it is observed that knockdown of FBL decreases the RSV M2–1 protein levels in HEP-2 cells. **(C)** Immunofluorescence showing RSV nucleoprotein (red) and nucleus (Blue). **(D)** Quantification of the percentage of RSV-infected cells under each condition. **(E, F)**. Furthermore, it is observed that knockdown increases the expression of the OAS1 and RIG-I. *P* values are determined by unpaired two-tailed *t*test. **P* < 0.05. All data are representative of three independent experiments and are presented as means ± s.d.

Supplementary Figure 3 Fibrillarin Restoration Promotes RSV Replication and Viral Protein Expression in HEP-2 Cells. **(A)** Exogenous fibrillarin expression in cells transfected with a fibrillarin-encoding plasmid. 24 hours post transfection. **(B)** RSV M2–1 protein levels in infected cells treated with control siRNA, FBL-targeting siRNA, or FBL siRNA followed by fibrillarin plasmid transfection. **(C)** Immunofluorescence showing RSV nucleoprotein (red) and fibrillarin (green) and nucleus (Blue). **(D)** Quantification of the percentage of RSV-infected cells under each condition. n.d (not detected). *P* values are determined by unpaired two-tailed *t*test. **P* < 0.05. All data are representative of three independent experiments and are presented as means ± s.d.

Supplementary Figure 4 Effects of fibrillarin silencing and restoration on RSV infection and ISG expression. **(A)** Analysis of ISG expression in HEP-2 Cells harvested 24 h post-transfection and 24 h post-infection under the same treatment conditions. Cells with fibrillarin restoration show a decrease in ISG levels compared with cells treated with FBL siRNA alone. n.d (not detected). P values were determined by unpaired two-tailed t-test. *P < 0.05. All data represent three independent experiments and are shown as means ± s.d.

Supplementary Figure 5 Silencing of the fibrillarin protein increases the expression of ISGS. **(A)** A549 cells transfected with siRNA FBL e infected with RSV at 1 MOI, where it is observed that knockdown of FBL decreases the infection in A549 cells. Furthermore, it is observed that knockdown increases the expression of the ISGs Oas1, Oas2, Ifiti3, PKR, DDX58 and RIG-I. *P* values are determined by unpaired two-tailed *t*test. **P* < 0.05. All data are representative of three independent experiments and are presented as means ± s.d.

Supplementary Table 1 Primers used for qRT-PCR
