# Supplementary figures and images for: Human respiratory syncytial virus regulates the expression of interferon-stimulated genes through modulation of fibrillarin
Source: Front Cell Infect Microbiol. 2026 Apr 15;16:1706028. doi: 10.3389/fcimb.2026.1706028 (PMC13125114; doi:10.3389/fcimb.2026.1706028)

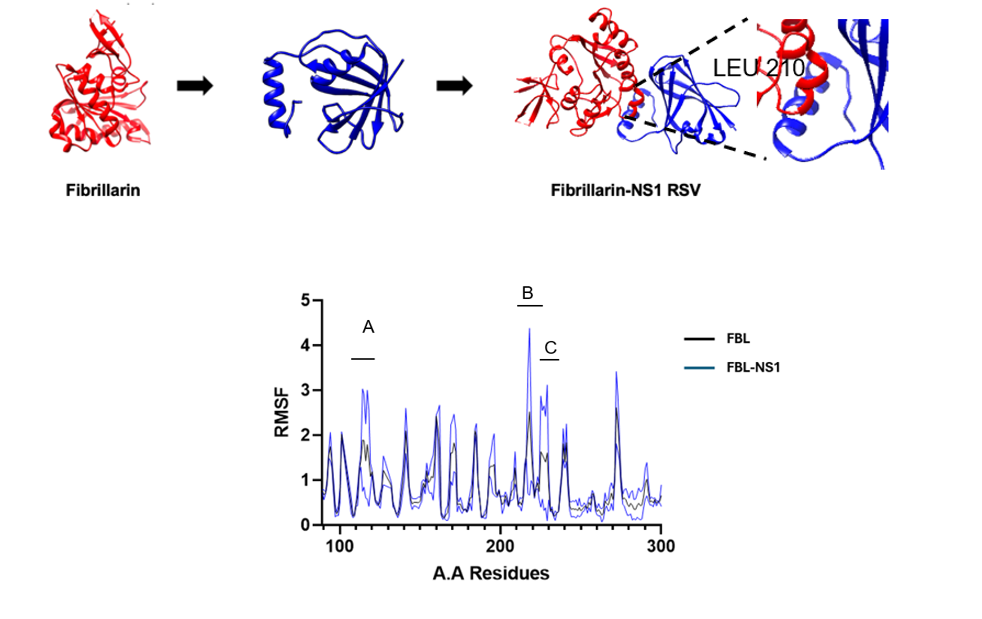

Supplement: Supplementary Figure 1 — In silico analysis to determine the interaction of RSV Non-Structural 1 protein with FBL cell protein. (A) Molecular docking analysis showing the three-dimensional representation of the respiratory syncytial virus (RSV) non-structural protein 1 and the fibrillarin protein. (B) Flexibility analysis of fibrillarin alone and in complex with the RSV non-structural protein 1, assessed by root mean square fluctuation (RMSF) of amino acid residues. Fibrillarin alone is shown in red, whereas fibrillarin in complex with the RSV non-structural protein 1 is shown in black. Higher RMSF values indicate regions with increased flexibility, while lower values correspond to more rigid regions. [file Image1.tif]

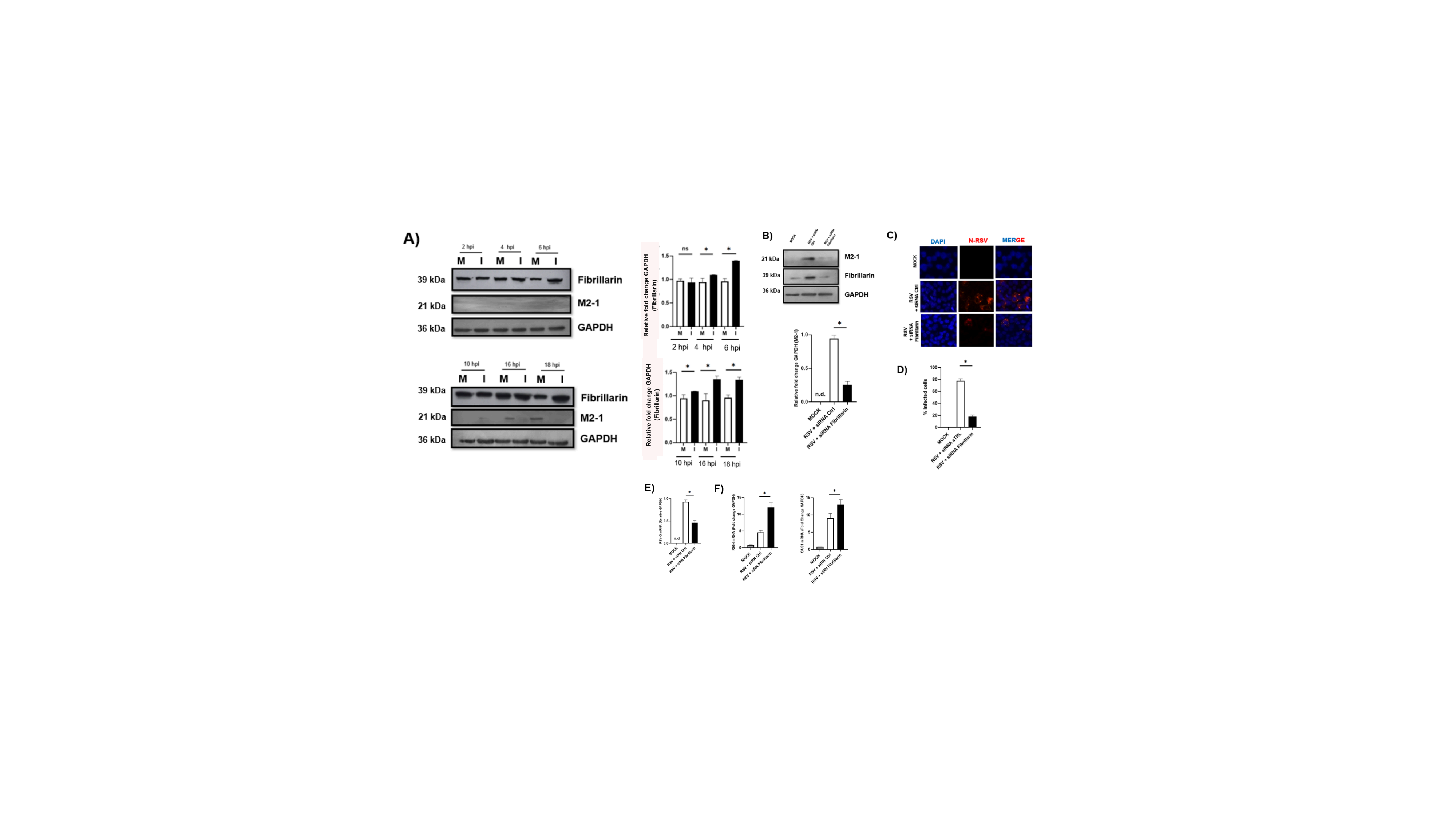

Supplement: Supplementary Figure 2 — RSV promotes FBL increase in early times of infection in HEP-2 cells. (A) Lysates of RSV- infected HEP-2 cells at 1 MOI that were analyzed by WB. It was observed that at the times 6 h, 10 h and 16 h and 18 h post-infection there is an increase in the FBL protein compared to the times 2 h and 4h h post-infection. (B) HEP-2 cells transfected with siRNA FBL e infected with RSV at 1 MOI, where it is observed that knockdown of FBL decreases the RSV M2–1 protein levels in HEP-2 cells. (C) Immunofluorescence showing RSV nucleoprotein (red) and nucleus (Blue). (D) Quantification of the percentage of RSV-infected cells under each condition. (E, F). Furthermore, it is observed that knockdown increases the expression of the OAS1 and RIG-I. P values are determined by unpaired two-tailed ttest. *P < 0.05. All data are representative of three independent experiments and are presented as means ± s.d. [file Image2.tiff]

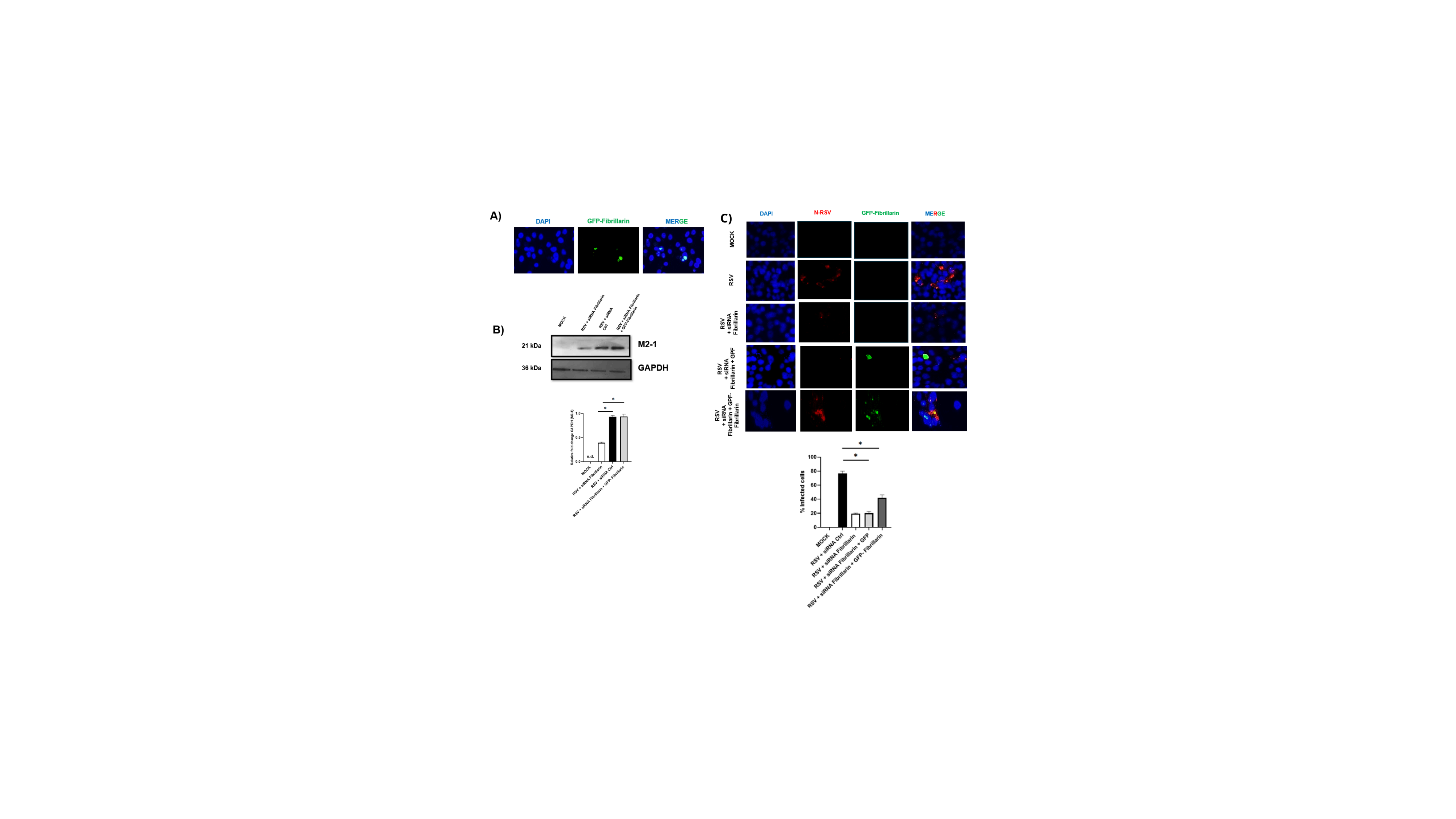

Supplement: Supplementary Figure 3 — Fibrillarin Restoration Promotes RSV Replication and Viral Protein Expression in HEP-2 Cells. (A) Exogenous fibrillarin expression in cells transfected with a fibrillarin-encoding plasmid. 24 hours post transfection. (B) RSV M2–1 protein levels in infected cells treated with control siRNA, FBL-targeting siRNA, or FBL siRNA followed by fibrillarin plasmid transfection. (C) Immunofluorescence showing RSV nucleoprotein (red) and fibrillarin (green) and nucleus (Blue). (D) Quantification of the percentage of RSV-infected cells under each condition. n.d (not detected). P values are determined by unpaired two-tailed ttest. *P < 0.05. All data are representative of three independent experiments and are presented as means ± s.d. [file Image3.tiff]

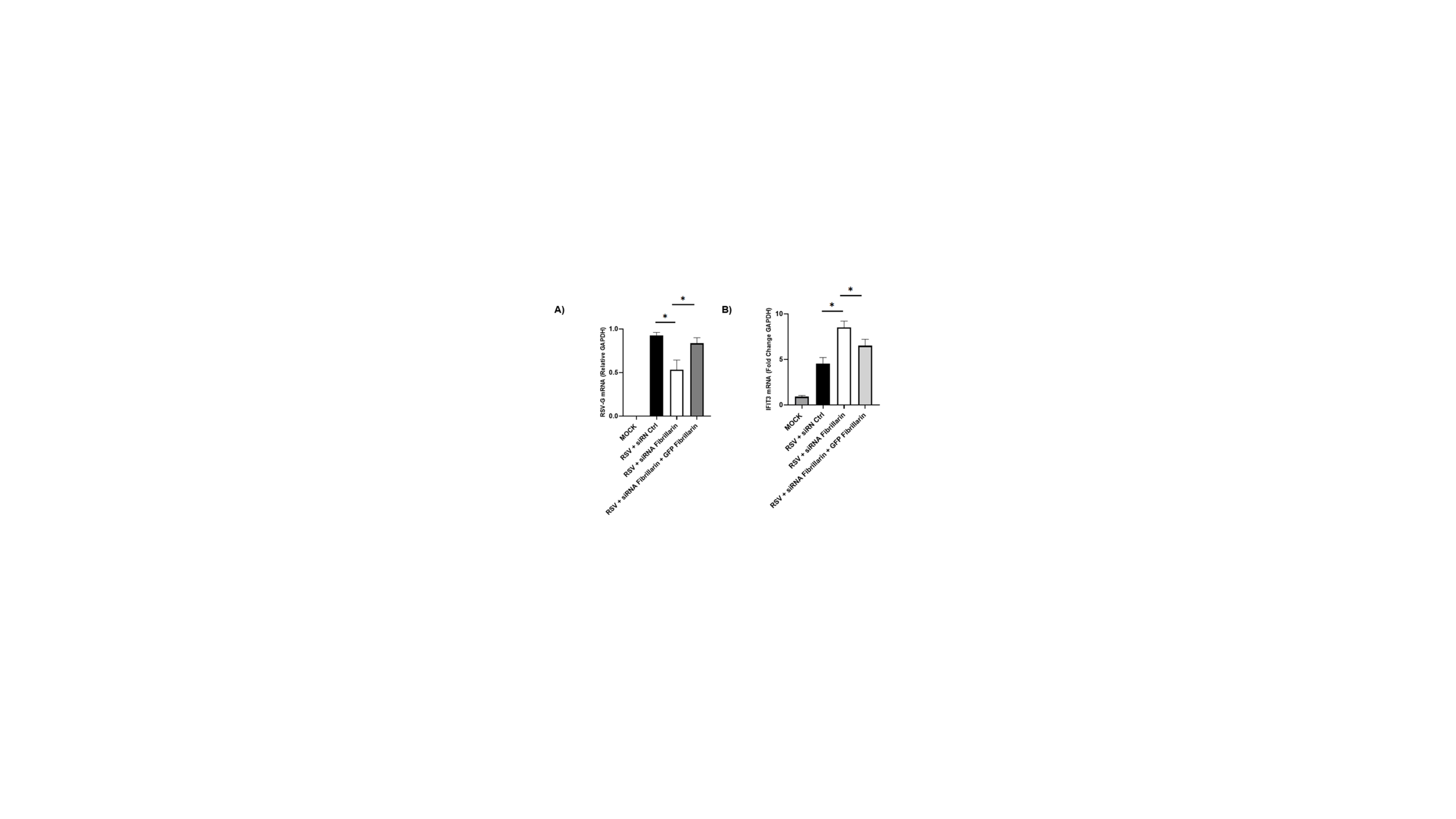

Supplement: Supplementary Figure 4 — Effects of fibrillarin silencing and restoration on RSV infection and ISG expression. (A) Analysis of ISG expression in HEP-2 Cells harvested 24 h post-transfection and 24 h post-infection under the same treatment conditions. Cells with fibrillarin restoration show a decrease in ISG levels compared with cells treated with FBL siRNA alone. n.d (not detected). P values were determined by unpaired two-tailed t-test. *P < 0.05. All data represent three independent experiments and are shown as means ± s.d. [file Image4.tiff]

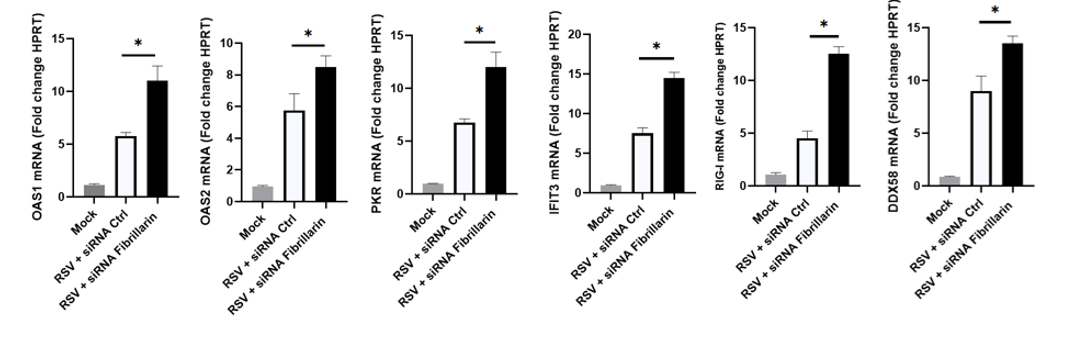

Supplement: Supplementary Figure 5 — Silencing of the fibrillarin protein increases the expression of ISGS. (A) A549 cells transfected with siRNA FBL e infected with RSV at 1 MOI, where it is observed that knockdown of FBL decreases the infection in A549 cells. Furthermore, it is observed that knockdown increases the expression of the ISGs Oas1, Oas2, Ifiti3, PKR, DDX58 and RIG-I. P values are determined by unpaired two-tailed ttest. *P < 0.05. All data are representative of three independent experiments and are presented as means ± s.d. [file Image5.tif]
